# Will County Medical Society

**Published:** 1875-02-01

**Authors:** 


EDITORS CHICAGO MEDICAL EXAMINER: — The Physicians and Surgeons of Will County, Illinois, met at the office of Dr. A. W. Heise, in Joliet, on January 5th, 1875, and organized "The Will County Medical

Society," by adopting a Constitution and By-Laws, in conformance with the Constitution of the State Medical Society.

Fourteen regular physicians became permanent members.

The officers elected for the current year, are A.W. Heise, M.D., of Joliet, President; E. R. Willard, M.D., of Wilmington, Vice-President; Censors, Geo. C. Raynor, M. D., of Joliet, Chas. H. Bacon, M.D., of Lockport, D. J. Merriman, M.D., of Wilmington; William Dougall, M.D., of Joliet, Secretary and Treasurer.

Drs. Speer and Abbott were elected Honorary Members.

Drs. Bacon and Mitchell were appointed to read essays at the next regular meeting.

Regular Meetings are held quarterly, in Joliet, on the first Tuesday of each quarter.

WILLIAM DOUGALL, M.D.,
*Secretary.*

JOLIET, Ill., Jan. 11, 1875.

